# Analysis of Change in the Wind Speed Ratio according to Apartment Layout and Solutions

**DOI:** 10.1155/2014/760713

**Published:** 2014-02-04

**Authors:** Won-gil Hyung, Young-Moon Kim, Ki-Pyo You

**Affiliations:** ^1^School of Architecture, Yeungnam University, Gyeongsan 712-749, Republic of Korea; ^2^Department of Architecture Engineering, Chonbuk National Uinversity, 567 Baekje-daero, Deokjin-gu, Jeonju-si, Jeollabuk-do 561-756, Republic of Korea

## Abstract

Apartment complexes in various forms are built in downtown areas. The arrangement of an apartment complex has great influence on the wind flow inside it. There are issues of residents' walking due to gust occurrence within apartment complexes, problems with pollutant emission due to airflow congestion, and heat island and cool island phenomena in apartment complexes. Currently, the forms of internal arrangements of apartment complexes are divided into the flat type and the tower type. In the present study, a wind tunnel experiment and computational fluid dynamics (CFD) simulation were performed with respect to internal wind flows in different apartment arrangement forms. Findings of the wind tunnel experiment showed that the internal form and arrangement of an apartment complex had significant influence on its internal airflow. The wind velocity of the buildings increased by 80% at maximum due to the proximity effects between the buildings. The CFD simulation for relaxing such wind flows indicated that the wind velocity reduced by 40% or more at maximum when the paths between the lateral sides of the buildings were extended.

## 1. Introduction

Recently, various damages due to winds blowing around buildings have occurred as low-rise, middle-rise, and high-rise buildings have been concentrated in downtown areas. Wind damage to buildings principally manifests in breach of the roof envelope, the wall envelope, and consequent damage to the building contents. Such damages are due to wind speed increase, most of which is influenced by surrounding buildings. Results from wind tunnel experiments building complexes with high-rise and low-rise buildings show that wind speed changes with high-rise buildings' sizes, low-rise buildings' heights, distances between buildings, and so on [1].

If a low-rise building is on the windward side of a high-rise building, the wind speed reaches its maximum due to the countercurrent flow almost in the middle position. In addition, the increase rate of wind speed reaches its maximum when the distance between a low-rise and a high-rise building is 0.5~1.0 time as long as the height of the latter. However, the increase rate of wind speed decreases when the distance between two buildings is less than a half of or longer than the height of the high-rise building [2]. Results from an experiment on valley wind blowing from between buildings show that the increase rate of wind speed reaches the maximum when the pitch of building is about 0.5~1.0 time as long as the width of the building [2]. Results from a wind tunnel experiment with respect to piloti show that the highest increase rate of wind speed appears in the central part of the pilotis when the height and width of a building are changed [2].

Though there have been many results from various wind tunnel experiments on wind speed changes around building surroundings, interpretations provided by most of them are centered on a single building or building groups consisting of 2 or 3 buildings. In addition, there is difficulty with performing lots of wind tunnel experiments due to modeling and the increase in experimental costs. Thus, currently, owing to the development of computers, wind environments of buildings are evaluated by means of the computational fluid dynamics (CFD) simulation. In computer simulations of CFD predictions, many interpretive elements (e.g., boundary conditions, interpretive domains, and lattice discretization) have a large influence on results. CFD studies on pedestrian levels of building surroundings with respect to each interpretive element are in progress [3–6]. Guidelines from some commercial CFD programs, as a method for verification of the effectiveness of CFD results, provide useful information for the analysis of airflows in building surroundings [7, 8]. However, such guidelines do not provide important information about pedestrian levels of building surroundings. CFD prediction guidelines for the evaluation of pedestrian levels of building surroundings are provided by the COST and Architectural Institute of Japan (AIJ) working groups [9, 10]. Using the results of such studies, a meta-study compared results from wind tunnel experiments and CFD results concerning the evaluation of wind environments in Japanese downtown areas. The comparison indicated that results from CFD simulations are reliable [11–13].

The present study selects three types of apartment complexes by means of the CFD simulation program tested by the AIJ working group and compared wind speed rates within the complexes in terms of wind tunnel experiments and CFD simulations. Then, it explores various methods of decreasing the wind speed ratio in the types of complexes in terms of CFD simulations.

## 2. Wind Tunnel Experiment

**Figure 1:**
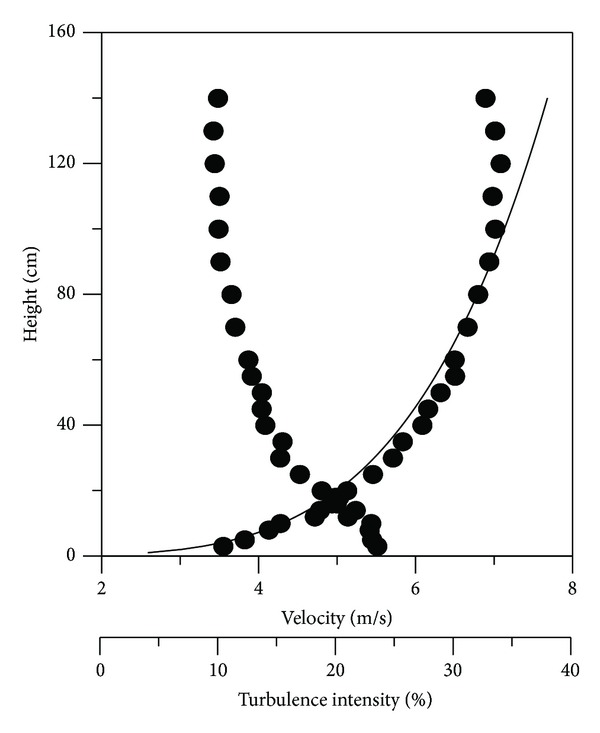
Vertical distribution of mean wind velocities and turbulence intensities.

The wind tunnel experiment was carried out with an open wind tunnel with 1.5 m (width) × 1.2 m (height) × 12 m (length) in the test section. Airflows in a turbulent boundary layer within a wind tunnel are simulated with ground roughness *B* (*α* = 0.22), as apartment complexes are placed generally in residential areas. The mean wind velocity variation with height is assumed to follow a power law, as given by (1). Turbulence intensity in each height was calculated by (2). The vertical distributions of the mean longitudinal wind velocities and turbulence intensities are shown in Figure 1

(1) $$\begin{array}{c} \frac{U\left(z\right)}{U\left(z_{r}\right)}={\left(\frac{z}{z_{r}}\right)}^{\alpha }, \end{array}$$

where *z* is height to yield, *z*_*r*_ is reference height, *U*(*z*) is wind speed at the height *z*, and *U*(*z*_*r*_) is wind speed at the reference height *z*_*r*_

(2) $$\begin{array}{c} \frac{\sigma_{u}}{U}=I_{ur}{\left(\frac{z}{z_{r}}\right)}^{-\alpha -0.05}, \end{array}$$

where *I*_*ur*_ is turbulence intensity at the reference height *z*_*r*_.

### 2.1. Experimental Models

Types of apartment arrangements are varied with forms of grounds and in accordance with planning laws; however, the present study used three representative types for the purposes of the experiment. The heights of the apartments were the same (60 m), and the models were produced in the same lot area and building coverage rate. The models were produced in the 1/400 scale with balsa materials. The experimental wind speed was 4 m/s at the 0.5 cm reference height (full scale: 2 m).

**Figure 2:**
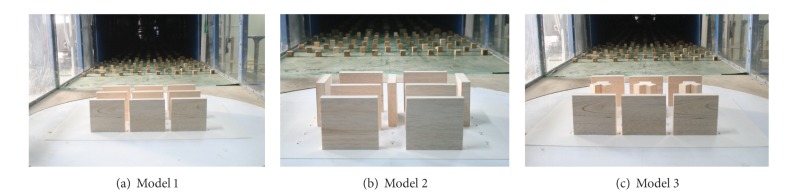
Experimental models.

Figure 2 shows the installation of the experimental models within the wind tunnel. Model 1 arranges most general flat-type apartments in parallel. Model 2 arranges flat-type apartments in a box form, and model 3 places tower-type apartments among flat-type ones.

**Figure 3:**
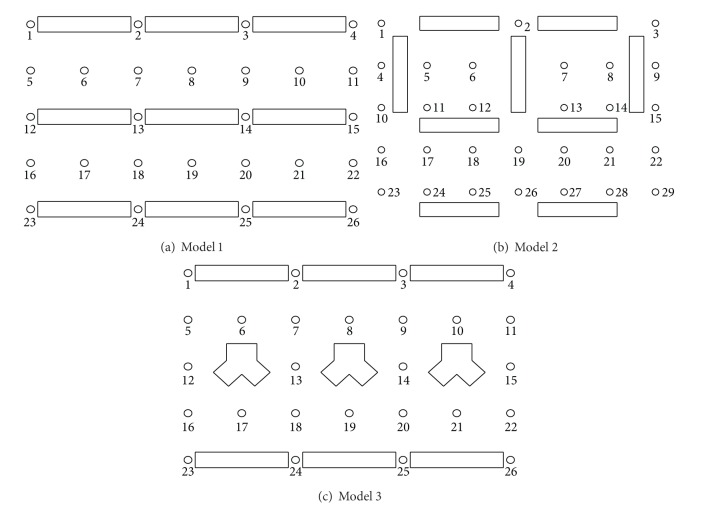
Measuring points.

The wind speed measuring points are shown in Figure 3. Wind speed was measured at 26 points in models 1 and 3, and 29 points in model 2. The measurement height was the same as the pedestrian level, 2 m, and the experiments were carried out in 16 wind directions with 22.5° intervals.

## 3. Computational Fluid Dynamics

Airflows around constructions are generally turbulent, with relatively high Reynolds number (Re). Turbulence includes various types, varying in severity. In the case of numerical simulation of turbulence, it is ideal to interpret all scales of vortexes by solving the basic equations of flows with the direct numerical simulation (DNS) method. However, as minute calculative lattices should be carried out in DNS in order to interpret all scales of vortexes, cases with high Re, like building surroundings, are not practical due to the high computational cost. In order to overcome this, methods are used that express only certain great scales of vortexes as objects of simulation. The standard *k*-*ε* model is a method to yield an ensemble average with a continuity equation (3) and a Navier-stokes equation (4) [14, 15]

(3) $$\begin{array}{c} \frac{\partial \langle u_{i}\rangle }{\partial x_{i}}=0, \end{array}$$

(4) ∂〈ui〉∂t+∂〈ui〉〈uj〉∂xj  =−1ρ∂〈p〉∂xi+∂∂xj[v∂〈ui〉∂xj−〈ui′uj′〉].

The most simple and basic model in Reynolds stress modeling −〈*u*_*i*_′*u*_*j*_′〉 is the eddy viscosity model. In analogy with the interaction formula for the shearing stress and velocity gradient generated by a coefficient of kinematic viscosity *ν*, eddy viscosity coefficient *v*_*t*_, and the Reynolds stress −〈*u*_*i*_′*u*_*j*_′〉 in relation to an average velocity gradient and *v*_*t*_,

(5) $$\begin{array}{c} -\langle u_{i}^{\prime }u_{j}^{\prime }\rangle =v_{t}\left[\frac{\partial \langle u_{i}\rangle }{\partial x_{j}}+\frac{\partial \langle u_{j}\rangle }{\partial x_{i}}\right]-\frac{2}{3}\delta_{ij}k=2\nu_{t}S_{ij}-\frac{2}{3}\delta_{ij}k, \end{array}$$

where *S*_*ij*_ = (1/2)[(∂〈*u*_*i*_〉/∂*x*_*j*_)+(∂〈*u*_*j*_〉/∂*x*_*i*_)], *k* = (1/2)〈*u*_*i*_′*u*_*i*_′〉, and *δ*_*ij*_: Kronecker delta.

The turbulent eddy viscosity *v*_*t*_ can be expressed as in (6) with the turbulence velocity scale *U* and the length scale *l*

(6) $$\begin{array}{c} v_{t}=l\cdot U. \end{array}$$

If the length scale of turbulence can be expressed as *l* = *U* · *t*_0_ with *U* the velocity scale and *t*_0_ the time scale, (6) can be expressed as (7)

(7) $$\begin{array}{c} v_{t}=U^{2}\cdot t_{0}. \end{array}$$

If the turbulence velocity scale *U* is the half square of the turbulence kinetic energy *k*, and the energy reduction rate *ε* can be evaluated as *k*/*ε*, this yields a relation of *v*_*t*_ ∝ *k* ^2^/*ε*. With a proportional constant *C*_*μ*_, *v*_*t*_ is yielded as in the following equation

(8) $$\begin{array}{c} v_{t}=C_{\mu }\frac{k^{2}}{\varepsilon }. \end{array}$$

The transfer equation of *k* is as in the following equation:

(9) $$\begin{array}{c} \frac{\partial k}{\partial t}+\langle u_{j}\rangle \frac{\partial k}{\partial x_{j}}=P_{k}+D_{K}-\varepsilon , \end{array}$$

where *P*_*k*_ can be defined as follows using (5):

(10) $$\begin{array}{c} P_{k}=-\langle u_{i}^{\prime }u_{j}^{\prime }\rangle \frac{\partial \langle u_{i}\rangle }{\partial x_{j}}=v_{t}\left[\frac{\partial \langle u_{i}\rangle }{\partial x_{j}}+\frac{\partial \langle u_{i}\rangle }{\partial x_{i}}\right]\frac{\partial \langle u_{i}\rangle }{\partial x_{j}}=v_{t}S^{2},\\ S=\sqrt{\frac{1}{2}{\left[\frac{\partial \langle u_{i}\rangle }{\partial x_{j}}+\frac{\partial \langle u_{j}\rangle }{\partial x_{i}}\right]}^{2}}=\sqrt{2S_{ij}S_{ij}},\\ D_{k}=\frac{\partial }{\partial x_{j}}\left[\frac{v_{t}}{\sigma_{k}}\cdot \frac{\partial k}{\partial x_{j}}\right]. \end{array}$$

With the transfer equation of *ε* yielded in (9),

(11) $$\begin{array}{c} \frac{\partial \varepsilon }{\partial t}+\langle u_{j}\rangle \frac{\partial \varepsilon }{\partial x_{j}}=\frac{\partial }{\partial x_{j}}\left[\frac{v_{t}}{\sigma_{\varepsilon }}\cdot \frac{\partial \varepsilon }{\partial x_{j}}\right]+\frac{\varepsilon }{k}\left(C_{\varepsilon 1}P_{k}-C_{\varepsilon 2}\varepsilon \right); \end{array}$$

the optimal variables can be expressed as [12]

(12) $$\begin{array}{c} \sigma_{k}=1.0,\;\;\sigma_{\varepsilon }=1.3,\;\;C_{\varepsilon 1}=1.44,\;\;C_{\varepsilon 2}=1.92. \end{array}$$

**Figure 4:**
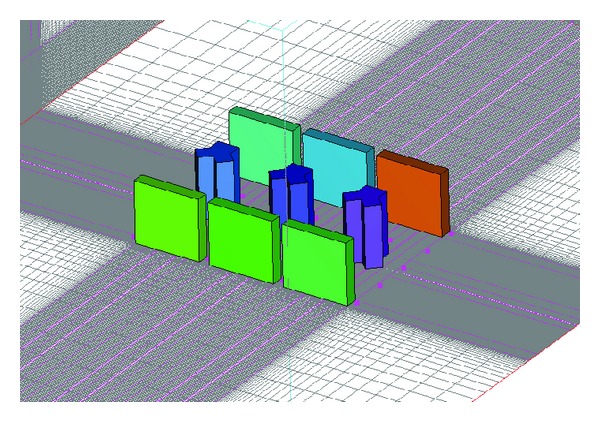
CFD modeling (model 3).

The CFD was performed to compare it with the wind speed ratios yielded from the wind tunnel experiment. The software used in this study is STREAM 9.0. The airflow was formed with *α* = 0.22 as in the wind tunnel experiment, and the turbulence model used the Standard *k*-*ε*. Other conditions, including the morphologies and arrangements of the buildings, were modeled in the same way as the wind tunnel experiment. The lattice simulation was carried out in the scope of about 1100 mm (*x*) × 900 mm (*y*) × 900 mm (*z*). The number of mesh segments was 100,000. The interpreted wind speed was identical to the experimental wind speed.  Figure 4 shows the CFD simulation modeling for model 3.

## 4. Result

The results of the experiments were represented in wind speed ratios, the definition of which is as in the following equation:

(13) $$\begin{array}{c} R_{i}=\frac{{\left(U_{h}\right)}_{i}}{U_{R}}, \end{array}$$

where (*U*_*h*_)_*i*_ is wind speed at point *i* (m/s) and *U*_*R*_ is wind speed of reference (m/s).

### 4.1. Wind Tunnel Experiment

**Figure 5:**
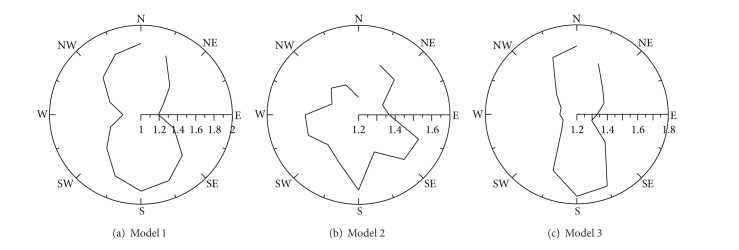
Distribution of the maximal wind speed ratio in each model.

The wind speed experiment was carried out in 16 wind angle directions. The wind speed is higher than the reference wind speed (4 m/s) with a wind speed ratio higher than 1, while it is as high as or lower than the reference wind speed, with a wind speed ratio of 1 or lower.  Figure 5 shows the maximal wind speed ratio in each wind angle in each model. The maximal wind speed was measured with north and south winds in models 1 and 3, and with south, west, and east winds in model 2. In the distribution of maximal wind speed ratios, wind speed ratio distributions of 1.5 or higher were frequent, in the order of model 1 > model 2 > model 3.

**Figure 6:**
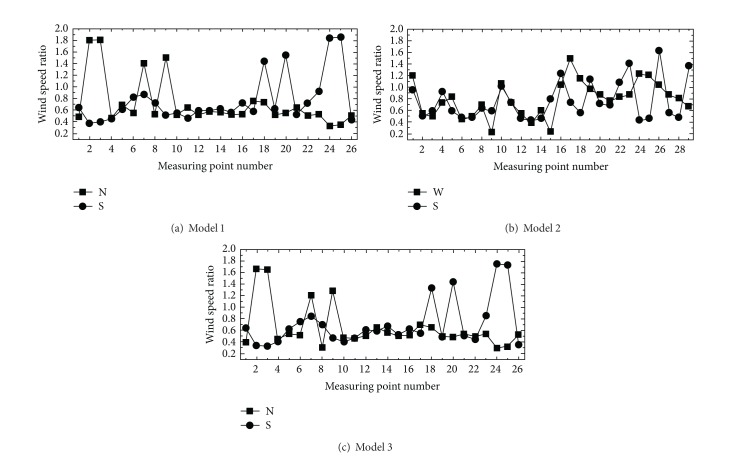
Wind speed ratio at each measurement point with respect to wind angle changes.

The wind speed ratios were greatly influenced by shapes and placements within the apartment complexes.  Figure 6 shows the wind speed ratio at each measurement point with respect to the wind angle at which the maximal wind speed ratio occurs in each model. In model 1, the wind speed ratios in measurement points 2 and 3 increased by 80% in the northern direction (N direction) of wind angle. This is consistent with the existing studies [5]. This is believed to be due to valley effects according to the pitch of building on the lateral and rear sides of the apartment placed in the direction of wind. The measurement is identical in the south wind angle. The pitch of building caused wind speed increased in straight placement. In model 2, with straight + box placement, the wind speed ratio increased by 60% as much (measurement point 26) in the south wind blowing from the straight placement as in the direction of wind blowing from the box-type placement. The mixed (rather than straight) placement influenced the wind speed within the apartment complexes. In addition, in model 3, where the apartments are arranged in a straight form outside, and in a tower form inside, the wind speed increased maximally by 60% at measurement points 2 and 3 and at points 24 and 25 between buildings in the north (N direction) and south (S direction) wind angles, respectively. Reduction in wind speed was maximally 20% in cases where the inner and outer buildings were arranged in different forms rather than in model 1 (where the inner and outer buildings were arranged in a uniform placement). The different forms between the inner and outer buildings of the complexes had effects on wind speed reduction. Such wind speed changes are due to the influence of residents' movement with the complexes.

### 4.2. Computational Fluid Dynamics

**Figure 7:**
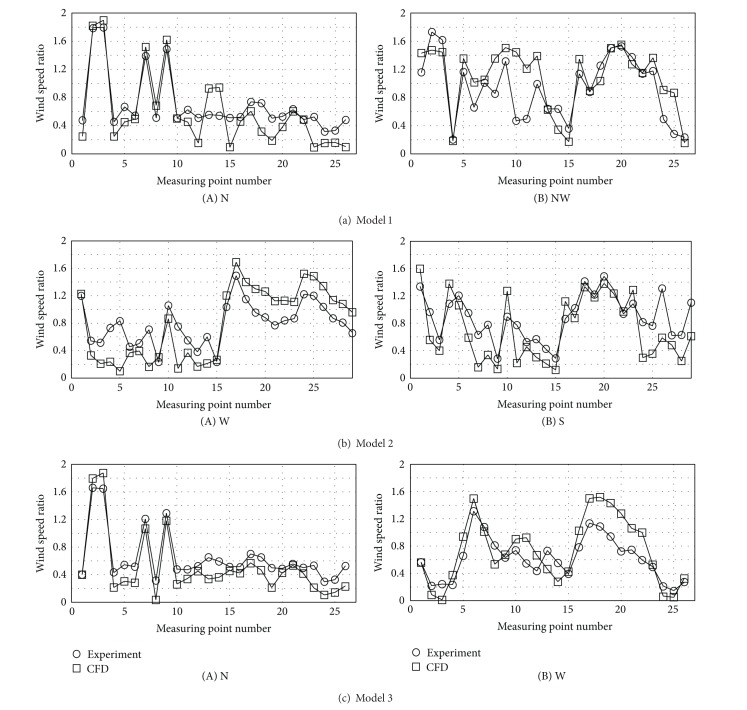
Comparison between the wind tunnel experiment and the CFD wind speed ratios at each measurement point.

**Figure 8:**
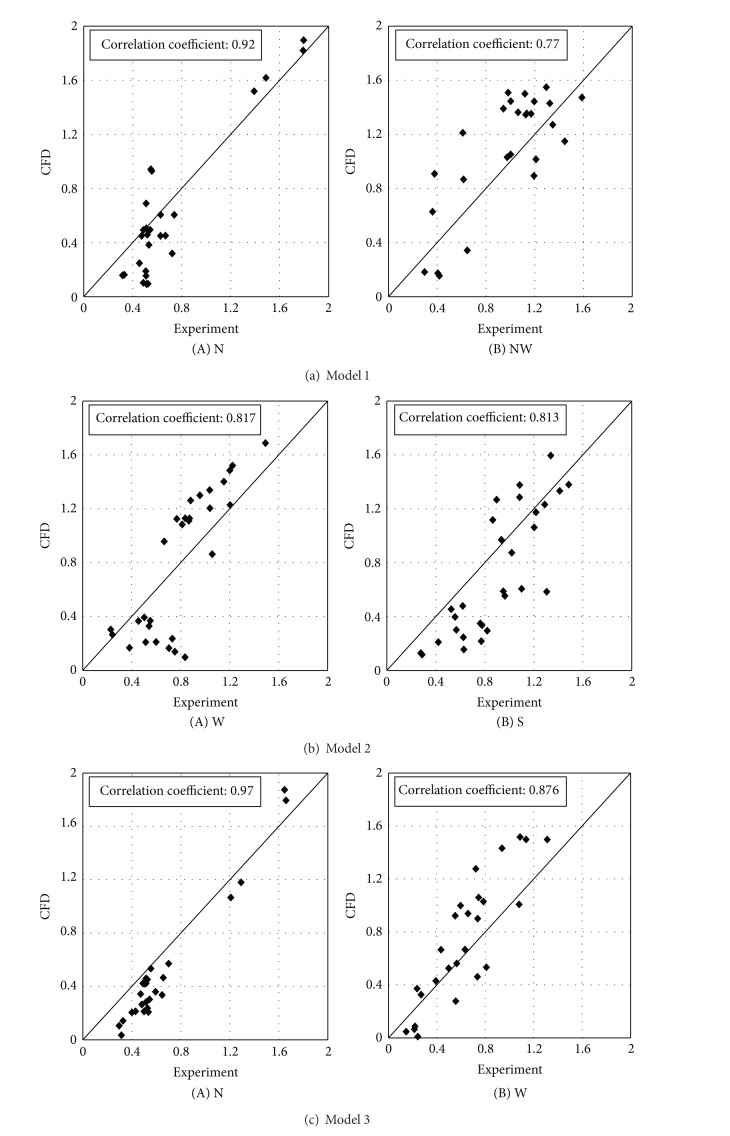
Correlation coefficients and standard errors of the wind speed experiment and CFD results in each case.

Various methods are used for leveling wind speed changes within complexes according to their placement types. The methods include wind speed decrease in terms of placement of buildings in varied angles and plantation of trees. As it takes much time and labor to perform various experiments for their evaluation, the present study tries to explore some improvements of wind environments of buildings in the CFD method. Such a study should be preceded by comparison between objects of the wind tunnel experiment and the CFD simulation.  Figure 7 is a comparison between the wind tunnel experiment and the CFD wind speed ratio at each measurement point in each experimental model. It compares them with focus on wind angles with great changes in wind speed ratio. The wind speed ratio changes at the measurement points placed in the direction of wind simulation in models 1 and 3 are almost consistent with the wind tunnel experiment and CFD results. However, there were differences (by 10~20%) from the wind tunnel experiment and CFD results on the rear sides of the buildings farthest from the direction of wind. This is believed to be influenced by the wake occurring between buildings. As the influence of the wake was less in the complexes with various placements than those with uniform placement (as in model 1), the distributions in models 2 and 3 were almost identical to the wind tunnel experiment and CFD results. The correlation coefficient in (14) was used to judge the relatedness between the wind speed ratios yielded from the wind tunnel experiment and the CFD. The correlation between the two wind speed ratios was stronger as the correlation coefficient got closer to 1, while they became more mutually independent as it approached 0.  Figure 8 yields the correlation coefficient between the wind tunnel experiment and the CFD in those wind angles with a great wind speed ratio. The correlation coefficient between the wind tunnel experiment and the CFD in each model was distributed in the range of 0.8~0.9. This suggests that the correlation between the CFD and the wind tunnel experiment is strong

(14) $$\begin{array}{c} R_{UV}=\frac{\sum_{i}^{n}U_{i}V_{i}-n\overset{-}{U}\;\overset{-}{V}}{\left(n-1\right)\sigma_{U}\sigma_{V}}, \end{array}$$

where *n* is number of samples, *U*, *V* are respective wind speed in the wind tunnel experiment and the CFD, and *σ* is standard deviation of wind speed.

## 5. Reducing Method

A general method of wind speed ratio reduction is to use landscapes. However, the present study involved changes to outer shapes of and distances between apartments. The north and the north-north-west directions were selected in model 1 for the simulation. Wind speed ratios were high in parts where the pitch of building side was narrowed and where wind flew out, hitting buildings. Method 1 places a pilotis on the ground floor of a building. Methods 2 and 3 make the lateral pitches of all the buildings increased 1.5 and two times, respectively, as high as the existing placement types. Method 4 makes the lateral pitches of the centers of the placed buildings increase by two times. This study performed its analysis only for north-north-west (NNW) wind angles, where the wind speed ratio was the highest in results of the wind tunnel experiment and CFD simulation in model 1.

**Figure 9:**
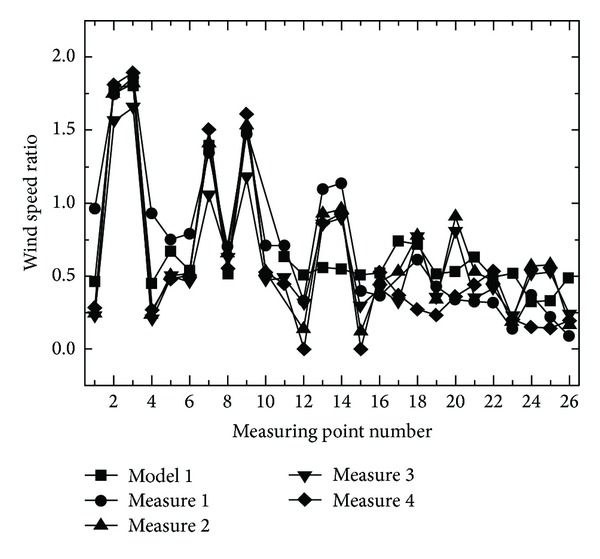
Distribution of the wind speed ratios at each measuring point based on the methods.

**Figure 10:**
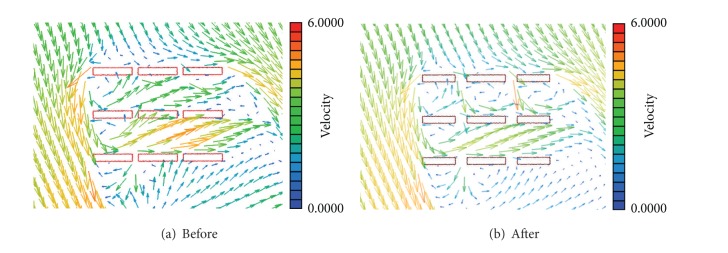
Results of the CFD simulation based on outer shape placement in model 1.

Figure 9 shows the wind speed ratios at each measurement point before and after the establishment of the reducing method for the NNW direction. In the NNW direction, extension of the pitch of building was more advantageous than the installment of pilotis in low floors. The reduction rates of wind speed ratios were the greatest, by 2%~61%, with measurement 4 for measurement points 18~21, where the greatest wind speed ratios were predicted before the measurement. The wind speed ratio was reduced at 14 measurement points in the case of method 2 and at 19 points in the case of method 3. The wider the paths between buildings, the less the wind speed ratios between apartments.  Figure 10 shows the results of the CFD simulation of the existing straight placement type for which the wind tunnel experiment was performed, and airflows in the vector form when the paths between buildings were increased by two times. All airflows weakened just with the securing of the paths between building sides, as in method 3.

## 6. Conclusions

The present study used three representative types of apartment complexes to compare wind speed ratios at each point of the complexes in terms of the wind tunnel experiment and the CFD simulation. In addition, it explored the effects on wind speed ratio reduction in each placement type and outer shape based on the CFD simulation.

1. As a result of the wind tunnel experiment based on apartment placement types and wind angle changes, it turned out that wind was greatly influenced by the inner forms and building placements within the apartment complexes. The rate of wind speed due to the adjacency effects between buildings increased by up to 80%. In particular, in cases like model 1 with straight placement, wind speed increased by 30~70% as much in the paths placed in the rear part of the complexes as in the paths which winds directly hit against the diagonal directions (NNW, SSW, SSE, and NNE).
2. The comparison between the results of the wind tunnel experiment and the CFD simulation showed rough consistency; however, the wind speed ratios yielded by the CFD simulation were higher than those yielded by the experiment, especially in those parts where some big wake occurred.
3. The CFD simulation in the N and NNW directions in the straight placement type (model 1) with the application of various methods for wind speed ratio reduction showed that the wind speed ratios were not reduced entirely as the direction of wind blowing faced the wide walls of apartments in the case of N, while it tended to show the greatest decrease overall when the paths between building sides were extended in the case of NNW.
